# Single vs. dual antiplatelet therapy before DOAC initiation in acute ischemic stroke with atrial fibrillation: a retrospective cohort study

**DOI:** 10.3389/fneur.2026.1882830

**Published:** 2026-08-06

**Authors:** Guan-xin Hou, Adili Tuersun, Zuo-jun Wang, Han Chen, Da-sheng Dang, Qing-chun Zhao, Yan Wang, Tian-shu Ren

**Affiliations:** 1 Department of Pharmacy, General Hospital of Northern Theater Command of PLA, Shenyang, China; 2 College of Clinical Pharmacy, Shenyang Pharmaceutical University, Shenyang, China; 3 State Key Laboratory of Advanced Drug Formulations for Overcoming Delivery Barriers, School of Pharmaceutical Sciences, Fudan University, Shanghai, China; 4 Department of Pharmacy, Dalian Rehabilitation Recuperation Center of Joint Logistics Support Force of PLA, Dalian, China

**Keywords:** acute ischemic stroke, antiplatelet therapy, bridging strategy, direct oral anticoagulants, functional outcome, non-valvular atrial fibrillation

## Abstract

**Background:**

The optimal antiplatelet bridging strategy before initiating direct oral anticoagulants (DOACs) in patients with acute ischemic stroke (AIS) and non-valvular atrial fibrillation (NVAF) remains uncertain.

**Methods:**

This single-center retrospective cohort study included 120 patients with AIS and NVAF who received antiplatelet therapy prior to DOAC initiation. Patients were stratified by regimen: single antiplatelet therapy (SAPT, *n* = 48) or dual antiplatelet therapy (DAPT, *n* = 72). Primary outcomes included early neurological improvement (≥2-point NIHSS reduction), early neurological deterioration (≥4-point NIHSS increase or death within 48 h), and in-hospital bleeding. Secondary outcomes were 90-day favorable functional outcome (modified Rankin Scale 0–2), 90-day excellent outcome (mRS 0–1), and ordinal mRS shift. Multivariate logistic regression identified independent predictors. Ordinal logistic regression was used for mRS shift analysis.

**Results:**

Baseline characteristics were comparable between groups, with no significant differences in baseline NIHSS score (median 4.00 vs. 2.00, *p* = 0.136) or mRS score (median 3.00 vs. 3.00, *p* = 0.435). No significant differences were observed in early neurological improvement (39.6% vs. 37.5%, *p* = 0.969), early deterioration (4.2% vs. 0%, *p* = 0.158), or bleeding events (6.2% vs. 1.4%, *p* = 0.301). In unadjusted analysis, DAPT demonstrated higher 90-day favorable outcome (90.3% vs. 75.0%, *p* = 0.046). However, after multivariable adjustment, DAPT was not independently associated with mRS 0–2 (adjusted OR 2.40, 95% CI 0.62–10.06, *p* = 0.211), mRS 0–1 (adjusted OR 1.18, 95% CI 0.43–3.14, *p* = 0.739), or ordinal mRS shift (adjusted common OR 0.70, 95% CI 0.34–1.44, *p* = 0.336). Baseline NIHSS score independently predicted all outcomes (*p* < 0.001 for all).

**Conclusion:**

In unadjusted analysis, DAPT prior to DOAC initiation was associated with higher 90-day favorable functional outcome without excess bleeding risk. However, this association was not independent of baseline stroke severity after multivariable adjustment, suggesting that the crude benefit was largely attributable to selection bias rather than a true therapeutic advantage of DAPT. The current data do not support the superiority of DAPT over SAPT. Baseline NIHSS score independently predicted both short-term and long-term outcomes. These hypothesis-generating findings warrant confirmation in adequately powered prospective randomized trials.

## 1 Introduction

Acute ischemic stroke (AIS) remains a leading cause of death and long-term disability worldwide, with its disease burden continuing to escalate in aging populations (1). Non-valvular atrial fibrillation (NVAF) constitutes the predominant etiological substrate for cardioembolic stroke, accounting for approximately 13 to 26% of all AIS cases (1). Compared with non-AF-related stroke, AF-related stroke is characterized by larger infarct volumes, more severe neurological deficits, and substantially higher early recurrence risk, with daily recurrence rates reaching 0.5 to 1.3% during the initial 2 weeks following symptom onset (2). Consequently, defining a safe and efficacious antithrombotic strategy within this high-risk temporal window represents a critical clinical imperative.

Anticoagulation therapy constitutes the guideline-directed cornerstone of secondary prevention for patients with AIS and comorbid NVAF. Nevertheless, the paradigm of early anticoagulation for cardioembolic stroke encountered substantial challenges prior to the widespread adoption of direct oral anticoagulants (DOACs). Landmark randomized controlled trials evaluating unfractionated or low-molecular-weight heparin—most notably the International Stroke Trial (IST) (3) and the Trial of ORG 10172 in Acute Stroke Treatment (TOAST) (4)—demonstrated that while early anticoagulation reduced recurrent ischemic events, this benefit was entirely offset by a significant excess of symptomatic intracranial hemorrhage (sICH), particularly among patients with large territorial infarcts or uncontrolled hypertension. Informed by these safety considerations, clinical guidelines and expert consensus coalesced around a deferred anticoagulation paradigm: initial antiplatelet therapy to safely navigate the acute phase, followed by delayed conversion to anticoagulation once clinical stabilization had been achieved.

Aspirin within 48 h of stroke onset is recommended by current guidelines (5), but its benefit in AF-related stroke is not statistically significant (subgroup analysis, IST and CAST) (6). Moreover, dual antiplatelet therapy (DAPT) has been shown to reduce recurrent stroke in patients with mild non-cardioembolic stroke (CHANCE (7), POINT (8) trials), but these trials excluded patients with AF. The ACTIVE-A trial (9) demonstrated that DAPT (aspirin plus clopidogrel) reduced major vascular events in patients with AF over a median follow-up of 3.6 years, yet evidence for early, short-term DAPT in AF-related stroke is absent. Even in the contemporary DOAC era, antiplatelet agents are routinely administered as bridging therapy during the “blank period” preceding DOAC initiation—a practice operationalized through decision rules such as the “1–3–6-12 day” algorithm (10) or the “4–7-14 day rule” (11)—in an effort to balance competing risks of early recurrent embolism and hemorrhagic transformation.

Contemporary investigations, including the TIMING (12), ELAN (13), and OPTIMAS (14) trials, have predominantly focused on optimizing the timing of anticoagulation initiation, generating robust evidence supporting the safety and efficacy of early DOAC commencement. However, a conspicuous evidence gap persists regarding the optimal antiplatelet bridging strategy—namely, whether single antiplatelet therapy (SAPT) or dual antiplatelet therapy (DAPT) should be preferred during the pre-anticoagulation interval. In pivotal trials such as ELAN and OPTIMAS, acute-phase antiplatelet therapy was not mandatory; for example, in OPTIMAS, 83.8% of participants received acute antiplatelet therapy before DOAC, but the specific regimen was not randomized. Thus, antiplatelet strategy selection for AIS patients with NVAF awaiting DOAC initiation represents a clinical decision node characterized by considerable uncertainty and a paucity of empirical guidance. The present real-world, retrospective study was designed to observe and compare outcomes of SAPT versus DAPT in this clinical context, not to prove superiority. It systematically compares SAPT versus DAPT with respect to early neurological trajectory, hemorrhagic complications, and long-term functional recovery, thereby providing preliminary evidence to inform clinical decision-making and establish baseline parameters for future prospective trial design.

## 2 Materials and methods

### 2.1 Database

This study utilized data derived from the electronic medical record database of the General Hospital of Northern Theater Command. The study protocol received approval from the Institutional Medical Ethics Committee (Approval No.: Y (2023) 123), with a waiver of informed consent granted in accordance with institutional policy governing retrospective analyses. Patients diagnosed with acute ischemic stroke complicated by non-valvular atrial fibrillation between January 1, 2019 and March 31, 2023 were identified through database query.

### 2.2 Study population

A total of 356 potentially eligible patients with NVAF and AIS were screened at the General Hospital of Northern Theater Command (a tertiary care center) during the study period. Following systematic application of prespecified inclusion and exclusion criteria, 120 patients were ultimately retained for analysis.

### 2.3 Inclusion criteria

Patients were considered eligible if they met all of the following criteria: (1) confirmed diagnosis of AIS with concomitant NVAF; (2) receipt of aspirin and/or clopidogrel antiplatelet therapy prior to anticoagulation initiation; and (3) subsequent treatment with a DOAC (rivaroxaban or dabigatran). Other DOACs (apixaban, edoxaban) were not available in the hospital formulary during the study period, and warfarin users were excluded due to the need for frequent INR monitoring and bridging with heparin, which would introduce additional confounding.

### 2.4 Exclusion criteria

Patients were excluded if any of the following conditions were present: (1) valvular atrial fibrillation or indeterminate valvular disease classification; (2) prior history of hemorrhagic stroke; (3) antecedent treatment with intravenous thrombolysis, mechanical thrombectomy, or endovascular intervention; (4) administration of argatroban for progressive stroke; (5) warfarin-based anticoagulation; (6) documented history of peptic ulcer disease, particularly with hemorrhagic complications, or active ulceration; (7) prior history of ischemic stroke; (8) incomplete imaging or laboratory data, or premature treatment discontinuation; (9) hospitalization duration less than 3 days without in-hospital anticoagulation initiation; (10) direct anticoagulation initiation without preceding antiplatelet therapy during the acute phase; and (11) loss to follow-up or failure to attend the 90 (±7)-day follow-up assessment.

### 2.5 Medications

#### 2.5.1 Anticoagulants

(1) Rivaroxaban tablets (Bayer Healthcare Pharmaceuticals, 20 mg per tablet).
(2) Dabigatran etexilate capsules (Boehringer Ingelheim Pharmaceuticals, 150 mg or 110 mg per capsule).

#### 2.5.2 Antiplatelet agents

(1) Aspirin enteric-coated tablets (Bayer Healthcare Pharmaceuticals, 100 mg per tablet).
(2) Clopidogrel bisulfate tablets (Sanofi [Hangzhou] Pharmaceuticals, 75 mg per tablet).

### 2.6 Study design and patient grouping

In this retrospective cohort study, 120 patients satisfying all inclusion and exclusion criteria were identified from the hospital electronic medical record database. Given the absence of established evidence regarding the optimal antiplatelet bridging strategy in this population, the choice between SAPT and DAPT was at the discretion of the treating neurologist, based on individual patient characteristics, including stroke severity, infarct size, and perceived bleeding risk, reflecting real-world clinical practice rather than a protocol-driven assignment. Patients were stratified into two groups according to the antiplatelet regimen administered prior to anticoagulation initiation: the SAPT group and the DAPT group. Beyond their assigned antiplatelet therapy, both groups received standard-of-care treatment directed at neuroprotection and circulatory optimization, alongside conventional rehabilitation comprising physical and occupational therapy.

#### 2.6.1 SAPT Group (*n* = 48)

Patients received single-agent antiplatelet therapy prior to anticoagulation initiation. The regimen consisted of aspirin 100 mg orally once daily, or clopidogrel 300 mg loading dose followed by 75 mg orally once daily as maintenance therapy.

#### 2.6.2 DAPT Group (*n* = 72)

Patients received dual antiplatelet therapy prior to anticoagulation initiation. The regimen consisted of aspirin 100 mg orally once daily combined with clopidogrel (300 mg loading dose followed by 75 mg orally once daily).

### 2.7 Baseline data collection

Comprehensive baseline characteristics were abstracted from the medical record for all enrolled patients at the time of index admission. Collected variables included age, sex, length of hospital stay, body mass index (BMI), alcohol consumption history, smoking history, National Institutes of Health Stroke Scale (NIHSS) score, modified Rankin Scale (mRS) score, and the presence of comorbid conditions including hypertension, diabetes mellitus, and coronary artery disease. These data were retrospectively extracted and subsequently analyzed to assess comparability between the SAPT and DAPT groups.

As part of the standard bridging strategy, antiplatelet therapy (aspirin with or without clopidogrel) was initiated immediately upon hospital admission following the diagnosis of acute ischemic stroke. The duration of antiplatelet therapy before transitioning to DOAC was guided by the “1–3–6-12 day” rule based on stroke severity and infarct size as assessed by the treating neurologist. Given the retrospective nature of this study, the exact timing from stroke onset to antiplatelet initiation, time from stroke onset to DOAC initiation, and total duration of antiplatelet therapy were not systematically documented in the medical records and could not be reliably ascertained for this retrospective analysis.

### 2.8 Outcome measures

#### 2.8.1 Primary efficacy and safety outcomes

(1) *Early neurological improvement*: Defined as a reduction in NIHSS score of ≥2 points from baseline to hospital discharge.
(2) *Early neurological deterioration*: Defined as an increase in NIHSS score of ≥4 points from baseline or death within 48 h of admission.
(3) *Bleeding events*: In-hospital occurrence of symptomatic intracranial hemorrhage, defined as any radiographic evidence of intracranial hemorrhage on head CT accompanied by clinically meaningful neurological deterioration (≥4-point increase in NIHSS score) as adjudicated by the study investigators or an independent safety monitor; major systemic bleeding, defined as a hemoglobin decline >2 g/dL, requirement for transfusion of ≥2 units of blood, or bleeding involving critical anatomical sites (e.g., gastrointestinal tract, retroperitoneum).

#### 2.8.2 Secondary outcomes

(1) *Favorable long-term functional outcome*: Defined as a 90-day mRS score of 0 to 2. Additionally, mRS 0–1 was analyzed as a more stringent secondary outcome.
(2) Distribution of 90-day mRS scores (ordinal analysis).
(3) *Recurrent vascular events at 90 days*: Incidence of recurrent ischemic stroke, myocardial infarction, or other vascular events within 90 days.
(4) *Mortality at 90 days*.

### 2.9 Statistical analysis

All statistical analyses were performed using R software version 4.5.3 (R Foundation for Statistical Computing, Vienna, Austria). Given that baseline NIHSS and mRS scores were not normally distributed (Shapiro–Wilk test *p* < 0.05), they are expressed as median and interquartile range [M (P₂₅, P₇₅)] and compared using the Mann–Whitney *U* test. Other continuous variables conforming to a normal distribution are expressed as mean ± standard deviation (SD) and compared using independent samples t-test. Categorical variables are summarized as frequencies and percentages [*n* (%)], with between-group comparisons executed using the Chi-square test or Fisher’s exact test when expected cell frequencies were less than 5.

Multivariate logistic regression analysis was employed to evaluate the independent effects of antiplatelet treatment strategy on early neurological improvement and 90-day favorable functional outcome (mRS 0–2), and mRS 0–1. Results are expressed as odds ratios (ORs) with corresponding 95% confidence intervals (CIs). Covariates included in the multivariable models comprised: antiplatelet treatment assignment (SAPT/DAPT), age, baseline NIHSS score, history of hypertension, and history of diabetes mellitus. These covariates were selected based on clinical relevance and established prognostic significance in stroke research, rather than on univariate *p*-value thresholds. Other baseline variables (BMI, smoking, alcohol consumption) were not included to avoid model overfitting given the limited sample size and number of outcome events. Ordinal logistic regression was used to analyze the shift in 90-day mRS distribution, with the common OR and 95% CI reported. Model goodness-of-fit was assessed using the Hosmer-Lemeshow test.

To explore potential heterogeneity of treatment effects across varying degrees of stroke severity, subgroup analyses were conducted stratifying patients by baseline NIHSS score into three categories: mild (0–4), moderate (5–15), and severe (≥16). These subgroup analyses are underpowered and should be interpreted with extreme caution. In logistic regression models, continuous variables including age and baseline NIHSS score were entered in their original continuous scale to preserve statistical power and avoid arbitrary categorization. Missing data were minimal across all analyzed variables (<5% for any given variable), and analyses were performed using complete cases without imputation. Sensitivity analyses were not performed, which is acknowledged as a limitation in the Discussion section.

Primary R packages utilized in this study included readxl (data import), dplyr (data manipulation), tableone (baseline table generation), ggplot2 (graphical rendering), and MASS (logistic regression analysis). All statistical tests were two-sided, with *p* < 0.05 considered statistically significant.

## 3 Results

### 3.1 Flowchart of patient selection

**Figure 1:**
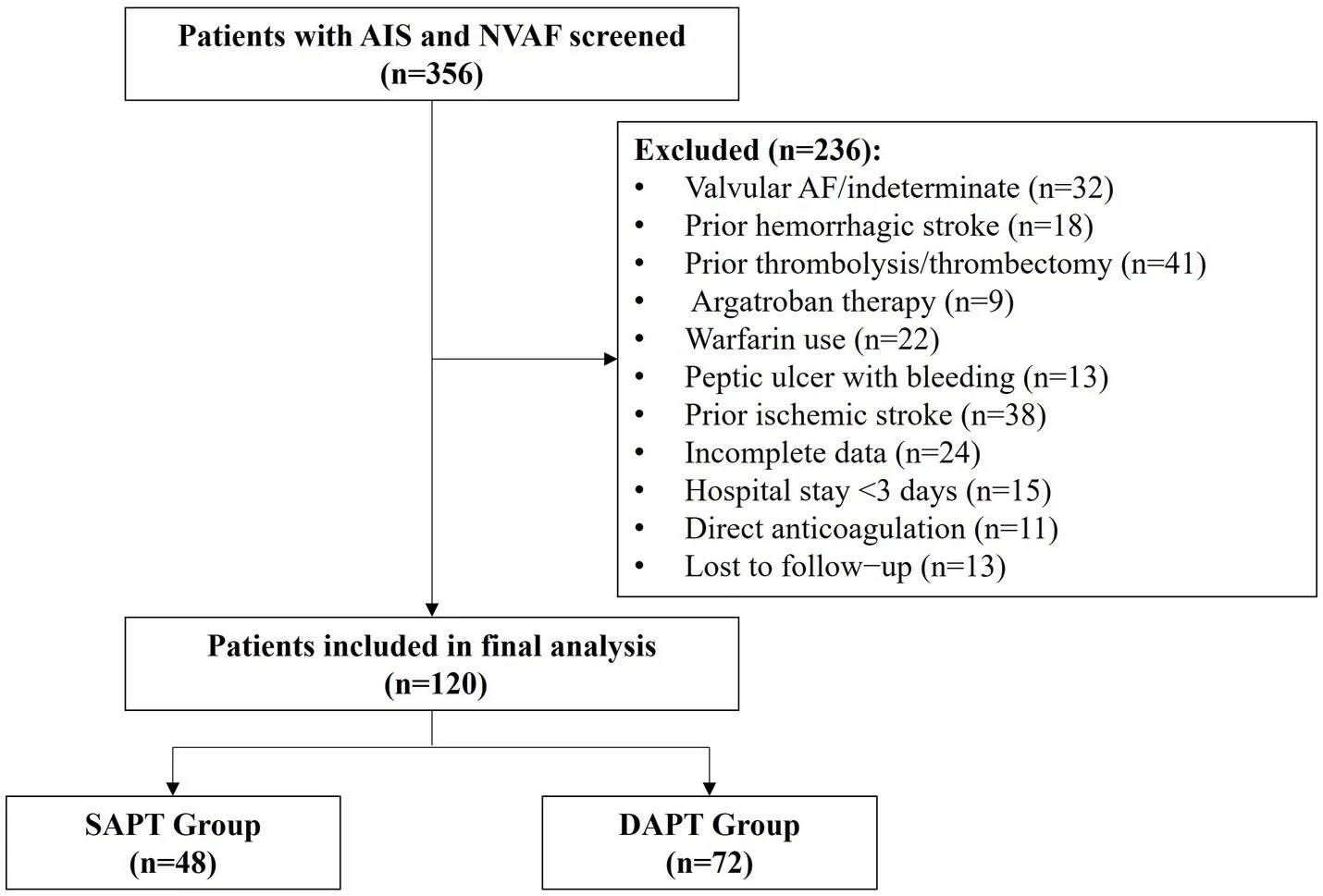
Flowchart of patient selection.

A total of 356 patients with acute ischemic stroke and non-valvular atrial fibrillation were screened from the hospital electronic medical record database between January 1, 2019 and March 31, 2023. After applying the prespecified exclusion criteria, 236 patients were excluded for the following reasons: valvular atrial fibrillation or indeterminate classification (*n* = 32), history of hemorrhagic stroke (*n* = 18), prior thrombolysis or thrombectomy (*n* = 41), argatroban therapy for progressive stroke (*n* = 9), warfarin anticoagulation (*n* = 22), history of peptic ulcer disease with bleeding (*n* = 13), prior ischemic stroke (*n* = 38), incomplete imaging or laboratory data (*n* = 24), hospitalization <3 days without in-hospital anticoagulation (*n* = 15), direct anticoagulation without preceding antiplatelet therapy (*n* = 11), and loss to follow-up at 90 days (*n* = 13). Consequently, 120 eligible patients were included in the final analysis and stratified according to pre-anticoagulation antiplatelet regimen: 48 patients in the SAPT group and 72 patients in the DAPT group (see Figure 1).

### 3.2 Baseline patient characteristics

A total of 120 patients with AIS and NVAF were included in the final analysis, comprising 48 patients in the SAPT group and 72 patients in the DAPT group. No statistically significant differences were observed between groups with respect to age, sex distribution, BMI, smoking, alcohol, or medical history (all *p* > 0.05). Baseline NIHSS score (median 4.00 [1.00–7.00] vs. 2.00 [1.00–4.25], *p* = 0.136) and mRS score (median 3.00 [2.00–4.00] vs. 3.00 [2.00–3.25], *p* = 0.435) were comparable between groups. Hospital stay was shorter in the DAPT group (9.62 ± 2.80 vs. 11.79 ± 5.55 days, *p* = 0.006) Detailed baseline characteristics are presented in Table 1.

**Table 1:** Comparison of baseline characteristics between groups at admission.

Characteristic	Overall (*n* = 120)	SAPT (*n* = 48)	DAPT (*n* = 72)	Test statistic	*p*-value
Demographics					
Age, years, mean ± SD	71.22 ± 10.40	72.17 ± 10.54	70.58 ± 10.34	*t* = 0.816	0.416
BMI, kg/m^2^, mean ± SD	24.16 ± 2.78	24.20 ± 2.64	24.13 ± 2.89	*t* = 0.136	0.892
Male sex, *n* (%)	66 (55.0)	25 (52.1)	41 (56.9)	χ^2^ = 0.114	0.736
Risk factors, *n* (%)					
Alcohol consumption, *n* (%)	31 (25.8)	14 (29.2)	17 (23.6)	χ^2^ = 0.219	0.640
Current smoking, *n* (%)	38 (31.7)	15 (31.2)	23 (31.9)	χ^2^ < 0.001	1.000
Medical history, *n* (%)					
Hypertension	81 (67.5)	35 (72.9)	46 (63.9)	χ^2^ = 0.698	0.403
Diabetes mellitus	25 (20.8)	8 (16.7)	17 (23.6)	χ^2^ = 0.475	0.491
Coronary artery disease	37 (30.8)	18 (37.5)	19 (26.4)	χ^2^ = 1.187	0.276
Clinical characteristics					
Hospital stay, days, mean ± SD	10.49 ± 4.24	11.79 ± 5.55	9.62 ± 2.80	*t* = 2.789	**0.006**
Baseline NIHSS score, median [IQR]	2.50 [1.00–6.00]	4.00 [1.00–7.00]	2.00[1.00–4.25]	*U* = 1482.5	0.136
Baseline mRS score, median [IQR]	3.00 [2.00–4.00]	3.00 [2.00–4.00]	3.00[2.00–3.25]	*U* = 1586.0	0.435

DAPT, dual antiplatelet therapy; IQR, interquartile range; mRS, modified Rankin Scale; NIHSS, National Institutes of Health Stroke Scale; SAPT, single antiplatelet therapy; SD, standard deviation.

Data are presented as mean ± SD for normally distributed continuous variables and median [IQR] for non-normally distributed continuous variables. Normally distributed continuous variables were compared using independent samples *t*-test; non-normally distributed variables were compared using Mann–Whitney *U* test. Categorical variables are presented as *n* (%) and compared using Chi-square test. Bold indicates *p* < 0.05.

### 3.3 Primary and secondary outcomes

Early neurological improvement (39.6% vs. 37.5%, *p* = 0.969), early deterioration (4.2% vs. 0%, *p* = 0.158 by Fisher’s exact test), and total bleeding events (6.2% vs. 1.4%, *p* = 0.301 by Fisher’s exact test) did not differ significantly (Table 2).

**Table 2:** Comparison of primary and secondary outcomes between groups.

Outcome	SAPT (*n* = 48)	DAPT (*n* = 72)	Test statistic	p-value
Primary efficacy outcomes				
Early neurological improvement, *n* (%)	19 (39.6)	27 (37.5)	0.001	0.969
Early neurological deterioration, *n* (%)	2 (4.2)	0 (0.0)	—	0.158^†^
Safety outcomes				
Total bleeding events, *n* (%)	3 (6.2)	1 (1.4)	—	0.301^†^
Intracranial hemorrhage, *n* (%)	1 (2.1)	0 (0.0)	—	0.400^†^
Gastrointestinal bleeding, *n* (%)	1 (2.1)	0 (0.0)	—	0.400^†^
Urinary tract bleeding, *n* (%)	1 (2.1)	0 (0.0)	—	0.400^†^
Other bleeding, *n* (%)	0 (0.0)	1 (1.4)	—	1.000^†^
Secondary outcomes				
90-day favorable outcome (mRS 0–2), *n* (%)	36 (75.0)	65 (90.3)	χ^2^ = 3.968	**0.046**
90-day excellent outcome (mRS 0–1), *n* (%)	26 (54.2)	48 (66.7)	χ^2^ = 1.412	0.235
90-day recurrent vascular events, *n* (%)	0 (0.0)	0 (0.0)	—	1.000^†^
90-day mortality, *n* (%)	2 (4.2)	1 (1.4)	—	0.563^†^

DAPT, dual antiplatelet therapy; mRS, modified Rankin Scale; SAPT, single antiplatelet therapy.

Data are presented as *n* (%). χ^2^ test was used for comparisons. ^†^Fisher’s exact test employed due to expected cell frequency <5 or small sample size; no χ^2^ statistic generated. Bold indicates *p* < 0.05.

After multivariable adjustment, antiplatelet strategy was not independently associated with mRS 0–2, mRS 0–1, or ordinal mRS shift. See Table 4 for detailed results.

**Figure 2:**
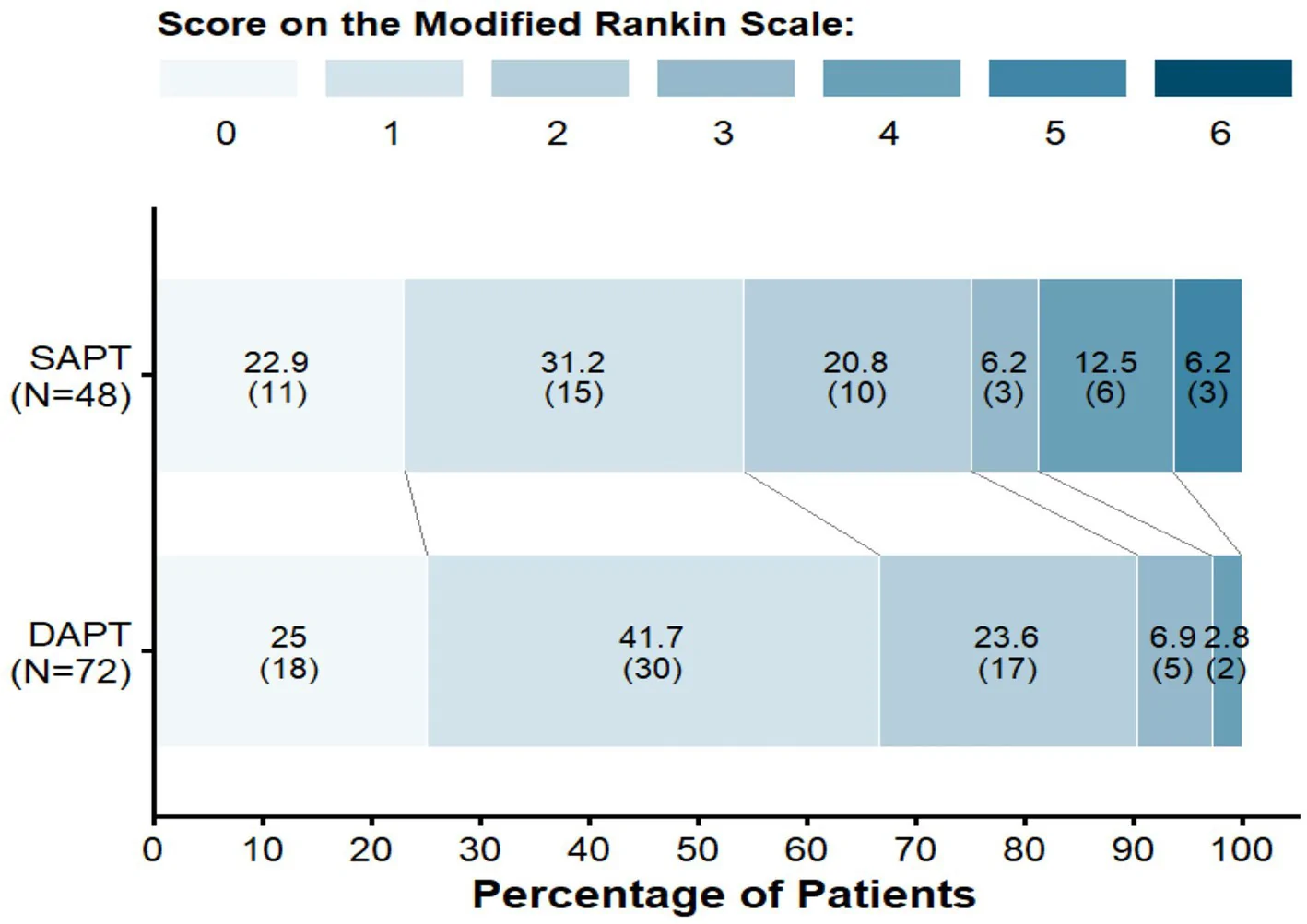
Distribution of 90-day modified Rankin Scale (mRS) scores. The Grotta bar graph displays the proportion of patients with each mRS score in the single antiplatelet therapy (SAPT, *n* = 48) and dual antiplatelet therapy (DAPT, *n* = 72) groups. The color gradient represents mRS scores ranging from 0 (lightest) to 6 (darkest). The overall distribution favored the DAPT group in the unadjusted analysis (Mann–Whitney *U* test, *p* = 0.025). The proportion of patients achieving an excellent outcome (mRS 0–1) was 54.2% in the SAPT group versus 66.7% in the DAPT group (unadjusted χ^2^ = 1.412, *p* = 0.235), and the proportion achieving a favorable outcome (mRS 0–2) was 75.0% versus 90.3% (unadjusted χ^2^ = 3.968, *p* = 0.046). However, after multivariable adjustment, antiplatelet strategy was not independently associated with any of these outcomes (see Table 4 for detailed results).

In unadjusted analysis, the DAPT group showed a higher rate of 90-day mRS 0–2 (90.3% vs. 75.0%, *p* = 0.046) and better mRS distribution (Mann–Whitney *U* test, *p* = 0.025). However, after multivariable adjustment, DAPT was not independently associated with mRS 0–2 (adjusted OR 2.40, 95% CI 0.62–10.06, *p* = 0.211), mRS 0–1 (adjusted OR 1.18, 95% CI 0.43–3.14, *p* = 0.739), or ordinal mRS shift (adjusted common OR 0.70, 95% CI 0.34–1.44, *p* = 0.336) (Tables 2, 4; Figure 2). No recurrent vascular events occurred. 90-day mortality was similar (4.2% vs. 1.4%, *p* = 0.563).

### 3.4 Multivariate logistic regression analysis

In multivariate logistic regression models, baseline NIHSS score emerged as the sole independent predictor of all outcomes, whereas antiplatelet strategy (DAPT vs. SAPT) was not independently associated with any endpoint after covariate adjustment (Tables 3, 4). For early neurological improvement, baseline NIHSS score was the only independent predictor (adjusted OR 1.48, 95% CI 1.28–1.76, *p* < 0.001). Antiplatelet strategy showed no independent association with early improvement (adjusted OR 1.74, 95% CI 0.67–4.85, *p* = 0.268) (Table 3).

**Table 3:** Multivariate logistic regression analysis of predictors for early neurological improvement.

Variable	*β* coefficient	SE	Wald	Adjusted OR (95% CI)	*p*-value
DAPT (vs. SAPT)	0.553	0.499	1.227	1.74 (0.67–4.85)	0.268
Age (per year)	0.009	0.022	0.177	1.01 (0.97–1.06)	0.674
Baseline NIHSS score (per point)	0.391	0.080	24.077	1.48 (1.28–1.76)	<0.001
Hypertension	0.106	0.499	0.045	1.11 (0.42–3.03)	0.831
Diabetes mellitus	−0.299	0.576	0.270	0.74 (0.23–2.22)	0.604

CI, confidence interval; DAPT, dual antiplatelet therapy; NIHSS, National Institutes of Health Stroke Scale; OR, odds ratio; SAPT, single antiplatelet therapy; SE, standard error. Hosmer-Lemeshow goodness-of-fit test: χ^2^ = 4.237, *p* = 0.835. Bold text denotes statistical significance (*p* < 0.05).

**Table 4:** Multivariate analysis of predictors for 90-day functional outcomes.

Variable	mRS 0–2 adjusted OR (95% CI)	*p-*value	mRS 0–1 adjusted OR (95% CI)	*p*-value	Ordinal mRS shift common OR (95% CI)	*p-*value
DAPT (vs. SAPT)	2.40 (0.62–10.06)	0.211	1.18 (0.43–3.14)	0.739	0.70 (0.34–1.44)	0.336
Baseline NIHSS score (per point)	0.62 (0.49–0.74)	<0.001	0.64 (0.52–0.75)	<0.001	1.63 (1.45–1.87)	<0.001

CI, confidence interval; DAPT, dual antiplatelet therapy; mRS, modified Rankin Scale; NIHSS, National Institutes of Health Stroke Scale; OR, odds ratio; SAPT, single antiplatelet therapy.

All models adjusted for age, hypertension, and diabetes mellitus. Hosmer-Lemeshow test for mRS 0–2: χ^2^ = 8.332, *p* = 0.402; for mRS 0–1: χ^2^ = 6.512, *p* = 0.590. Bold text denotes *p* < 0.05.

For 90-day functional outcomes, three complementary analyses were performed. Baseline NIHSS independently predicted favorable outcome defined as mRS 0–2 (adjusted OR 0.62, 95% CI 0.49–0.74, *p* < 0.001), excellent outcome defined as mRS 0–1 (adjusted OR 0.64, 95% CI 0.52–0.75, *p* < 0.001), and ordinal mRS shift (adjusted common OR 1.63, 95% CI 1.45–1.87, *p* < 0.001) (Table 4). In contrast, DAPT was not independently associated with any of these outcomes: mRS 0–2 (adjusted OR 2.40, 95% CI 0.62–10.06, *p* = 0.211), mRS 0–1 (adjusted OR 1.18, 95% CI 0.43–3.14, *p* = 0.739), or ordinal mRS shift (adjusted common OR 0.70, 95% CI 0.34–1.44, *p* = 0.336). The discrepancy between the significant unadjusted association and the non-significant adjusted association for mRS 0–2 suggests that the crude benefit of DAPT was largely attributable to differences in baseline stroke severity rather than a true therapeutic advantage.

### 3.5 Subgroup analysis

Given the limited sample size, these subgroup analyses are exploratory in nature and should be interpreted with extreme caution. Subgroup analysis stratified by baseline NIHSS score severity revealed no statistically significant differences in early neurological improvement rates between the SAPT and DAPT groups across any severity stratum (all *p* > 0.05). With respect to 90-day favorable functional outcome, numerically higher rates were consistently observed in the DAPT group within both mild (94.2% vs. 82.1%, *p* = 0.129) and moderate (78.9% vs. 66.7%, *p* = 0.480) stroke strata, although these differences did not achieve statistical significance. However, given the limited sample size, particularly in the moderate and severe strata, these negative findings should not be interpreted as evidence of equivalence between SAPT and DAPT.

Meaningful statistical comparison was precluded in the severe stroke stratum owing to the limited sample size (n = 3). Interaction testing revealed no significant interaction between treatment assignment and baseline stroke severity for either early neurological improvement or 90-day favorable outcome, but these interaction tests are also grossly underpowered in the current sample.

In summary, while the direction of effect favored DAPT across all strata, the small sample sizes—especially in the severe stratum (*n* = 3)—preclude any meaningful subgroup conclusions. These analyses are presented for descriptive and hypothesis-generating purposes only. These findings are summarized in Table 5.

**Table 5:** Subgroup analysis of outcomes stratified by baseline NIHSS score.

NIHSS strata	Group	*n*	Early Neurological Improvement	*p*-value	90-day favorable outcome	*p*-value
Mild (0–4)	SAPT	28	8 (28.6%)	1.000	23 (82.1%)	0.129
	DAPT	52	15 (28.8%)		49 (94.2%)	
Moderate (5–15)	SAPT	18	9 (50.0%)	0.744	12 (66.7%)	0.480
	DAPT	19	11 (57.9%)		15 (78.9%)	
Severe (≥16)	SAPT	2	2 (100.0%)	—^†^	1 (50.0%)	—^†^
	DAPT	1	1 (100.0%)		1 (100.0%)	

DAPT, dual antiplatelet therapy; NIHSS, National Institutes of Health Stroke Scale; SAPT, single antiplatelet therapy.

*p*-values derived from Fisher’s exact test for within-strata comparisons. ^†^Statistical testing not performed due to small sample size (*n* = 3) in this stratum.

Important Caveat: These subgroup analyses are exploratory and grossly underpowered (NIHSS strata: mild *n* = 80, moderate *n* = 37, severe *n* = 3). The severe stroke stratum (*n* = 3) cannot support any meaningful statistical inference. These results should be interpreted with extreme caution and are presented for descriptive purposes only. No treatment-by-severity interaction was observed, but interaction tests are also underpowered in this sample.

## 4 Discussion

This single-center retrospective cohort study systematically compared the efficacy and safety of single versus dual antiplatelet therapy administered prior to DOAC initiation in patients with acute ischemic stroke and non-valvular atrial fibrillation. The principal findings are threefold: (1) no statistically significant differences were observed between groups with respect to early neurological improvement or hemorrhagic complications; (2) in unadjusted analysis, the DAPT group exhibited a significantly higher rate of 90-day favorable functional outcome, but this association disappeared after multivariable adjustment; (3) multivariate logistic regression analysis identified baseline NIHSS score as the sole independent predictor of all outcomes. The current data do not support the superiority of DAPT over SAPT, and the observed unadjusted benefit is likely attributable to confounding by indication.

### 4.1 Early neurological trajectory and bleeding risk

Early neurological improvement rates were comparable between the SAPT and DAPT groups (39.6% vs. 37.5%, respectively, *p* = 0.969). This observation suggests that during the abbreviated bridging interval preceding DOAC initiation, the intensity of antiplatelet therapy—whether single- or dual-agent—exerts minimal influence on the short-term neurological recovery trajectory during the acute hospitalization period. Early neurological evolution in cardioembolic stroke is predominantly governed by factors including infarct core volume, adequacy of collateral circulatory compensation, and the occurrence of early reperfusion, rather than by antiplatelet regimen intensity per se. Notably, baseline NIHSS score emerged as an independent positive predictor of early improvement in multivariable analysis (adjusted OR 1.48, 95% CI 1.28–1.76, *p* < 0.001), reflecting the clinically intuitive phenomenon whereby patients with more severe initial deficits possess greater “room for improvement.” This should not, however, be misconstrued as indicative of favorable ultimate prognosis.

Regarding safety, total bleeding event rates were numerically higher in the SAPT group relative to the DAPT group (6.2% vs. 1.4%, *p* = 0.301). This counterintuitive finding is most plausibly explained by selection bias: patients in the DAPT group exhibited numerically lower baseline NIHSS scores and significantly shorter hospital stays, collectively suggesting milder overall disease burden and inherently lower bleeding risk. The absolute number of bleeding events was limited (*n* = 4 overall), yielding constrained statistical power; consequently, the absence of a statistically significant between-group difference should not be construed as evidence of equivalent bleeding risk. Notwithstanding these considerations, the complete absence of intracranial hemorrhage in the DAPT group provides preliminary reassurance regarding the short-term safety of DAPT bridging in carefully selected patients. Extant literature similarly indicates that short-term (≤21 days) DAPT does not substantially elevate hemorrhagic transformation risk in patients with minor ischemic stroke (7).

### 4.2 Between-group differences in 90-day functional outcomes

The observation that DAPT was associated with a significantly higher rate of 90-day favorable functional outcome (90.3% vs. 75.0%, *p* = 0.046) constitutes the most clinically salient finding of this investigation. However, this association was attenuated and failed to retain statistical significance following multivariable adjustment for age, baseline NIHSS score, and comorbid conditions (adjusted OR 2.40, 95% CI 0.62–10.06, *p* = 0.211). To further examine the robustness of this finding, two complementary analyses were performed: mRS 0–1 (excellent outcome) and ordinal mRS shift analysis. In both analyses, DAPT was not independently associated with outcome (mRS 0–1: adjusted OR 1.18, 95% CI 0.43–3.14, *p* = 0.739; ordinal mRS: adjusted common OR 0.70, 95% CI 0.34–1.44, *p* = 0.336), reinforcing the conclusion that the crude association was driven by confounding rather than a true therapeutic effect.

This pattern—a significant unadjusted association that disappears after adjustment—is a hallmark of confounding by indication. Clinicians may have preferentially allocated patients with less severe stroke and lower perceived bleeding risk to DAPT, while reserving SAPT for those with more severe presentations or heightened bleeding concerns. The trends toward lower baseline NIHSS scores and significantly shorter hospital stays in the DAPT cohort support this inference. It is therefore important to explicitly state that the current data do not support the superiority of DAPT over SAPT.

The potential biological rationale for DAPT—enhanced protection against microembolic events from non-cardioembolic sources, and synergistic platelet inhibition in the acute post-stroke period (15)—remains speculative. The adjusted OR of 2.40, while not statistically significant, may inform sample size calculations for future confirmatory trials.

### 4.3 Prognostic utility of baseline NIHSS score

Multivariable analyses consistently demonstrated that baseline NIHSS score independently predicts both early neurological improvement (adjusted OR 1.48, *p* < 0.001) and 90-day favorable functional outcome (adjusted OR 0.62, 95% CI 0.49–0.74, *p* < 0.001), mRS 0–1 (adjusted OR 0.64, *p* < 0.001), and ordinal mRS shift (adjusted common OR 1.63, *p* < 0.001). These findings align closely with the existing evidentiary base, reaffirming the central role of NIHSS score as a prognostic instrument in stroke populations (16). The directionally opposite associations observed for the two outcome measures—whereby higher baseline scores predict greater likelihood of early improvement yet lower probability of ultimate favorable recovery—reflect a clinically coherent paradox: patients with higher NIHSS scores possess greater capacity for measurable score reduction during the acute phase, yet their more severe initial neurological impairment renders complete functional recovery (mRS 0–2) substantially more difficult to achieve.

### 4.4 Insights from subgroup analyses

Subgroup analyses stratified by baseline NIHSS severity revealed numerically higher 90-day favorable outcome rates with DAPT across both mild (94.2% vs. 82.1%) and moderate (78.9% vs. 66.7%) stroke strata, although these differences did not achieve statistical significance. However, these analyses are grossly underpowered, particularly the severe stroke stratum (*n* = 3), and should be interpreted with extreme caution. The absence of a significant treatment-by-severity interaction should not be interpreted as evidence of consistent effects across strata, as interaction tests are also underpowered. Future studies should prioritize investigation of optimal antithrombotic strategies in this high-risk population.

### 4.5 Comparison with existing literature

Our study addresses a critical evidence gap. While TIMING (12), ELAN (13), and OPTIMAS (14) focused on the timing of DOAC initiation, the present study provides the first real-world systematic comparison of the antiplatelet bridging strategy itself. In these pivotal trials, acute-phase antiplatelet therapy was not mandatory, and the specific regimen was not randomized. DAPT is an established standard for acute non-cardioembolic minor stroke (CHANCE (7), POINT (8)), but its role in cardioembolic stroke remains unproven, as these trials excluded patients with AF. The ACTIVE-A trial (9) demonstrated long-term benefit of DAPT in AF patients, but evidence for early, short-term bridging is absent. Our findings, while negative for DAPT superiority, provide important real-world data on current prescribing patterns and outcomes, highlighting the need for randomized evidence in this specific clinical context. While ACTIVE-A established the long-term efficacy of DAPT in AF patients, our study extends this inquiry to the acute bridging phase, finding no clear advantage of DAPT over SAPT once stroke severity is accounted for.

### 4.6 Limitations

Several limitations of this investigation warrant acknowledgment.

First, the retrospective observational design is inherently susceptible to selection bias and residual confounding. Treatment assignment was determined by attending physician discretion rather than randomization, potentially introducing confounding by indication. Although multivariable regression analysis was employed to adjust for age, baseline NIHSS score, and major comorbid conditions, unmeasured confounders—including infarct volume, left atrial appendage morphology, and socioeconomic status—may have influenced the observed outcomes.

Second, the study is constrained by limited sample size and inadequate statistical power. As a retrospective analysis, sample size was dictated by the number of eligible patients meeting inclusion and exclusion criteria during the study period rather than prospective power calculations. Post-hoc power analysis for the primary endpoint of 90-day favorable functional outcome—with observed rates of 75.0% in the SAPT group and 90.3% in the DAPT group (absolute difference 15.3%)—indicates that the current sample (48 SAPT, 72 DAPT; total n = 120) achieved approximately 48.7% power at a two-sided *α* = 0.05. This falls substantially below the conventional 80% threshold, implying an elevated risk of type II error (false-negative rate approximately 51.3%). Similarly, statistical power for low-frequency endpoints such as bleeding events (*n* = 4 total) is severely limited; consequently, the absence of a statistically significant between-group difference (*p* = 0.301) should not be construed as evidence of equivalent bleeding risk. In addition, propensity score matching was not performed, as the limited sample size precluded meaningful matched analyses without substantial loss of statistical efficiency. Notwithstanding these power limitations, the effect size estimates derived from this study (absolute benefit 15.3%, adjusted OR 2.40) provide critical parameters to inform sample size calculations for future confirmatory randomized controlled trials.

Third, the single-center setting may restrict generalizability to other healthcare environments, demographic populations, and practice patterns.

Fourth, important clinical variables were unavailable. Constrained by the retrospective design, we were unable to collect data on infarct volume, radiographic hemorrhagic transformation subtype, left atrial dimensions and appendage morphology, post-discharge DOAC adherence, and concomitant medication use (e.g., proton-pump inhibitors), and precise timing from stroke onset to antiplatelet initiation and DOAC transition-all factors with potential implications for functional outcomes and bleeding risk.

Fifth, the follow-up duration was relatively abbreviated. While a 90-day assessment window aligns with conventional benchmarks in stroke clinical trials, it is insufficient to evaluate long-term functional recovery trajectories, delayed vascular event recurrence, or late hemorrhagic complications.

Sixth, recurrent vascular event data were conspicuously absent. No recurrent ischemic strokes or myocardial infarctions were documented in either group during the 90-day follow-up period, which may be attributable to limited sample size, abbreviated follow-up, survival bias, or incomplete event capture. This finding should therefore be interpreted with caution.

## 5 Conclusion

This retrospective cohort study suggests that among patients with AIS and NVAF, the initiation of DAPT prior to DOAC administration was associated with a higher rate of 90-day favorable functional outcome in unadjusted analysis (90.3% vs. 75.0%), without an increase in observed bleeding events. However, this association did not maintain statistical significance after multivariable adjustment, indicating that the crude benefit was largely attributable to baseline differences in stroke severity rather than a true therapeutic advantage of DAPT. The current data do not support the superiority of DAPT over SAPT.

Baseline NIHSS score independently predicted both early neurological improvement and long-term functional recovery, including mRS 0–2, mRS 0–1, and ordinal mRS shift, reaffirming its central prognostic role in this patient population.

Given the inherent limitations of the retrospective design, limited sample size (post-hoc power of 48.7%), and potential for residual confounding, these findings should be regarded as hypothesis-generating. The absence of excess bleeding with DAPT provides preliminary reassurance for its short-term safety during the pre-anticoagulation window, but the absence of recurrent vascular events in both groups should be interpreted with caution. Future rigorously designed, large-scale prospective randomized controlled trials are warranted to definitively establish the optimal antiplatelet bridging strategy prior to anticoagulation initiation in patients with atrial fibrillation-associated stroke.

## Data Availability

The data analyzed in this study is subject to the following licenses/restrictions: The datasets analyzed in this study are subject to institutional data privacy policies and ethical restrictions related to patient medical records. Data are available from the corresponding author upon reasonable request, subject to approval by the Institutional Medical Ethics Committee. Requests to access these datasets should be directed to T-sR, sz_pharm@163.com.
